# Overactive Bladder during Pregnancy: A Prospective Longitudinal Study

**DOI:** 10.3390/medicina58020243

**Published:** 2022-02-06

**Authors:** Hung-Ju Chen, Sheng-Mou Hsiao, Cheng-Fang Yang, Chien-Nan Lee, Yi-Wen Wang, Da-Wei Guo, Shiow-Ru Chang

**Affiliations:** 1 Department of Nursing, Tri-Service General Hospital, Taipei 114, Taiwan; r05426007@ntu.edu.tw; 2 Women’s Health & Maternal-Newborn Nursing Program, School of Nursing, College of Medicine, National Taiwan University, Taipei 100, Taiwan; r02426009@ntu.edu.tw; 3 Department of Obstetrics and Gynecology, Far Eastern Memorial Hospital, Banqiao, New Taipei 220, Taiwan; smhsiao2@gmail.com; 4 Department of Obstetrics and Gynecology, National Taiwan University College of Medicine and the Hospital, Taipei 100, Taiwan; leecn@ntu.edu.tw; 5 Graduate School of Biotechnology and Bioengineering, Yuan Ze University, Taoyuan 320, Taiwan; 6 Second Degree Bachelor of Science, College of Medicine, National Taiwan University, Taipei 100, Taiwan; fionayang@ntu.edu.tw; 7 Department of Nursing, National Taiwan University Hospital, Yunlin Branch, Yunlin 640, Taiwan; 8 Institute of Emergency and Critical Care Medicine, College of Medicine, National Yang Ming Chiao Tung University, Taipei 112, Taiwan; evavictor2001@gmail.com; 9 School of Nursing, College of Medicine, National Taiwan University, Taipei 100, Taiwan; 10 Department of Nursing, National Taiwan University Hospital, Taipei 100, Taiwan

**Keywords:** overactive bladder, pregnancy, prevalence

## Abstract

*Background and Objectives:* Overactive bladder (OAB) is a serious urination-related symptom of unknown pathogenesis that affects one’s everyday activities. The objective of this study was to examine how OAB prevalence, symptom severity, and degree of distress caused by OAB symptoms evolved throughout the course of pregnancy. *Materials and Methods:* A total of 659 pregnant women were recruited from 2015 to 2020, and were evaluated through the International Consultation on Incontinence Questionnaire-Overactive Bladder (ICIQ-OAB) on OAB symptoms, administered in the early, middle, and late stages of pregnancy. *Results:* Generalized estimating equation analysis revealed that the odds of OAB occurring in the middle and late stages of pregnancy were 1.90 and 2.33 times higher, respectively, than in early pregnancy. The corresponding odds for OAB-wet were 1.63 and 2.07 higher, respectively, and the odds of OAB-dry occurring during late pregnancy were 0.80 higher than during early pregnancy. Symptoms were more severe by 0.07 and 0.21 points (on a 4-point scale) in the middle and late stages of pregnancy, respectively, than in early pregnancy; distress was greater by 0.13 and 0.27 points (on a 10-point scale) in the middle and late stages of pregnancy, respectively, than in early pregnancy. The prevalence of OAB, OAB-dry, and OAB-wet was significantly higher in early pregnancy than pre-pregnancy. *Conclusions:* The prevalence of OAB and OAB-wet increased over the course of pregnancy, but the prevalence of OAB-dry decreased. Furthermore, symptom severity and degree of distress increased over time.

## 1. Introduction

According to the International Continence Society (ICS), overactive bladder (OAB), usually accompanied by increased urination during the day or nocturia, is a serious urination-related symptom that occurs without urinary tract infection or other diagnosable diseases. OAB with and without the symptom of urge urinary incontinence is called OAB-wet and OAB-dry, respectively [1]. The pathogenesis of OAB remains unclear. At present, two main pathogeneses (neurogenic and myogenic) have been proposed regarding OAB’s complex pathophysiological changes [2]. The symptoms relating to OAB during pregnancy are due to normal physiological changes, and resolve following delivery [3].

OAB affects a patient’s everyday life, including their work, travel, physical movements, social interactions, sleep, and even their sexual function [4]. Women are particularly affected, and they tend to avoid hobbies where urinary leaks or the lack of a toilet can pose a problem [5]. For women, OAB also affects their mental health, resulting in depression, anxiety, embarrassment, low self-esteem, and poor sleep quality, negatively influencing their quality of life and relationships [6]. Studies have indicated that approximately 25% of women encounter difficulties in experiencing sexual arousal, orgasm, and pleasure [7]. Those with severer OAB symptoms have a significantly lower quality of life [8]. A survey involving first-time mothers who were 36 weeks pregnant revealed that OAB-wet, but not OAB-dry, significantly influences their quality of life with respect to their everyday activities, bodily functions, social interactions, emotional state, and sense of embarrassment [9].

In 2001, Milsom et al. conducted a survey in six European countries, and discovered that 16.6% of the citizens aged 40 years and above had OAB symptoms [10]. In 2003, Stewart et al. also conducted a census in the U.S. and reported that 16.5% of citizens aged 18 years and above had OAB symptoms, among whom 6.1% had OAB-wet and 10.4% had OAB-dry [11]. In Asia, the prevalence of OAB in Japan is low, at 8.1% [12], whereas the prevalence of OAB in China, North Korea, and Taiwan among citizens aged 40 years was high, at 23.9%, 19.7%, and 15.8%, respectively [8]. Few studies, however, have focused on OAB among pregnant women in western countries [9,13,14]. However, studies have not used the OAB classification of the International Continence Society in their analysis. Moreover, no study in eastern countries has analyzed OAB in pregnant women. Thus, in our study, we examined the prevalence, symptom severity, and degree of distress of OAB among pregnant women, and observed how their symptoms evolved throughout the course of pregnancy.

This study tested the hypothesis that OAB prevalence, symptom severity, and degree of distress caused by OAB symptoms would increase over time throughout pregnancy.

## 2. Materials and Methods

We recruited women who were in the early stages of pregnancy from an obstetrics outpatient department of a medical center in Northern Taiwan between 2015 and 2020. Data relating to OAB and the participants’ background were gathered throughout the early, middle, and late stages of pregnancy when the patients went for prenatal examination. A participant was included if they were (1) older than 20 years of age, (2) in early pregnancy (<17 weeks of pregnancy) and had received a pregnancy booklet, and (3) literate in traditional Chinese and lucid. A prospective participant was excluded if they (1) exhibited signs of miscarriage, (2) had a urinary tract infection, or (3) could not fill out the research consent form and questionnaire. This project was approved by the Research Ethics Committee of the hospital involved. All participants signed an informed consent declaration.

### 2.1. Data Collection

We explained our research aims and process to prospective participants visiting the hospital’s outpatient obstetrics department for prenatal examination. The inclusion criteria were women ≥20 years old and during early pregnancy, and the subjects provided their written informed consent prior to their participation. Women who reported urinary tract abnormalities or urinary tract infection in need of regular medical follow-up were excluded from the study population. Participants could withdraw from the study at any time, and their medical rights were not compromised regardless of whether they participated or withdrew. Because follow-up visits were required, the participants gave their contact details on the consent form. We informed them that only the researchers would contact them using their phone number or address. The information provided was for research use only, and all information was kept confidential in order to protect the patients’ right to privacy. To track the condition of their pregnancy, the participants completed another questionnaire when they revisited the outpatient obstetrics department for prenatal examination during middle (17–28 weeks of pregnancy) and late pregnancy (≥29 weeks of pregnancy). Furthermore, participants were also invited to recall their experience of OAB during pre-pregnancy. In total, 961 eligible participants remained after ineligible participants were screened. Among the 961 participants, 46 refused to participate, and 26 did not complete the questionnaire; in addition, 729 and 659 participants went for middle and late pregnancy follow-up visits, respectively. In total, 197 participants withdrew or refused to participate; the rejection and participant loss rates were 20.5% and 10.9%, respectively.

### 2.2. Measurement

We employed two measurement tools: a sheet on basic demographic information, and a self-developed structured questionnaire that measured basic demographic attributes and obstetric variables. The demographic attributes measured were age, body mass index (BMI), level of education, occupation, and household income. The obstetric variables were number of weeks of pregnancy, number of pregnancies, and birth history.

We applied the Taiwan (i.e., traditional Chinese) version of the international consultation on incontinence questionnaire, overactive bladder module (ICIQ-OAB). The ICIQ-OAB is a self-administered questionnaire assessing the following four OAB symptoms: frequency, nocturia, urgency, and urge urinary incontinence [15]. The ICIQ-OAB comprises six items, where the first and second items concern the date of birth and gender, respectively. The third to sixth items are individually divided into two sub-items a and b. Items 3a, 4a, 5a, and 6a pertain to the severity of the four symptoms on a 5-point Likert scale (0 to 4 points), while items 3b, 4b, 5b, and 6b pertain to the degree of distress caused by the symptom, measured using a visual analog scale (0–10 points), with higher scores indicating a more distressing symptom. In the four preceding weeks, patients who urinated 7–8 times/day (3a ≥ 1 point) were defined as having frequent urination; those who reported waking up to urinate twice during the night (4a ≥ 2 points) were defined as having nocturia; those who reported having “occasionally” feel that they “have to rush to the toilet” (5a ≥ 1 point) or “urinate before reaching the toilet” (6a ≥ 1 point) were defined as having urgency or urge urinary incontinence, respectively. The total score for the four sub-items ranges from 0–16 points, with a higher score indicating increased symptom severity. Urgency in urination was defined as a necessary symptom for OAB, along with at least one other symptom. OAB with and without the occurrence of urge urinary incontinence is referred to as OAB-wet and OAB-dry, respectively. The definitions of symptoms are listed in Table 1.

### 2.3. Statistical Analysis

We used SPSS for Mac v 25 for statistical analysis, and set the significance level to be *p* < 0.05. The basic demographic attributes and obstetric variables were analyzed using descriptive statistics. Continuous variables (e.g., symptom severity and degree of symptom distress) were expressed in terms of the mean and standard deviation, while discrete variables (e.g., OAB prevalence from early to late pregnancy) were expressed in terms of frequency and percentage. In addition, a generalized estimating equation (GEE) was adopted for repeated measurements in order to estimate the changes in OAB prevalence, symptom severity, and degree of symptom distress.

## 3. Results

### 3.1. Sample Characteristics

In total, 889, 729, and 659 women completed valid questionnaires during early, middle, and late pregnancy, respectively. The data of 659 participants were analyzed. The distributions of the demographic variables are detailed in Table 2. The mean age was 33.82 (SD = 4.13). The mean durations during early, middle, and late pregnancy were 12.54 (SD = 2.73), 23.90 (SD = 3.28), and 33.61 (SD = 2.74) weeks, respectively. The mean weights in early, middle, and late pregnancy were 57.77 (SD = 9.82), 62.35 (SD = 9.90), and 66.57 (SD = 10.03), respectively. The mean BMI values in early, middle, and late pregnancy were 22.43 (SD = 3.64), 24.18 (SD = 3.65), and 25.84 (SD = 3.71), respectively.

### 3.2. Changes in OAB throughout Pregnancy

**Figure 1:**
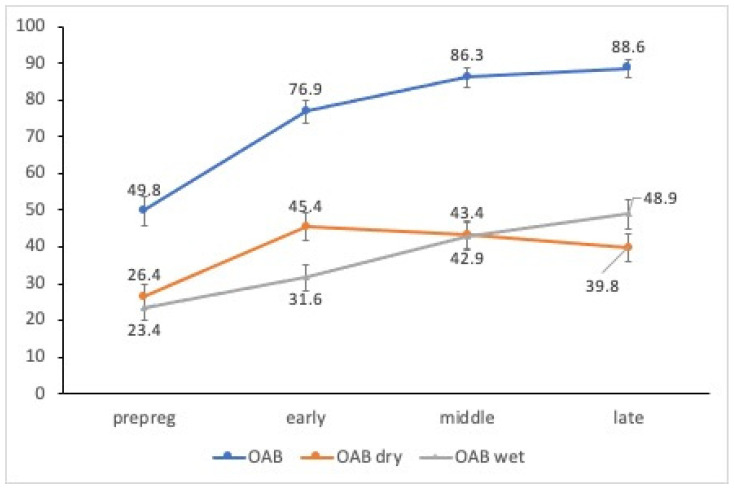
Prevalence of OAB, OAB-dry, and OAB-wet during early, middle, and late pregnancy.

We found that the prevalence of OAB and OAB-wet increased over time from pre-pregnancy to late pregnancy, while the prevalence of OAB-dry increased from pre-pregnancy to early pregnancy, and decreased in the middle and late stages (Figure 1). We used a GEE to examine the changes in the prevalence of OAB during pregnancy, and inspected the influence of time on the prevalence of OAB, as shown in Table 3. In the results, the odds of OAB occurring in middle and late pregnancy were 1.90 and 2.33 times higher, respectively, than in early pregnancy (*p* < 0.001). The post hoc polynomial test verified that the trend was linear, at statistical significance (*p* < 0.001), and thus corroborated the finding that the prevalence of OAB (especially OAB-wet) during late pregnancy was significantly higher than during early and middle pregnancy. The odds of OAB-wet occurring in middle and late pregnancy were 1.63 and 2.07 times higher, respectively, than in early pregnancy (*p* < 0.001), meaning that OAB-wet becomes significantly more prevalent during late pregnancy.

The prevalence of OAB-dry, however, exhibited a different trend, increasing in early pregnancy and decreasing thereafter. We conducted GEE analysis of the changes in the prevalence of OAB-dry, and analyzed the influence of time on the prevalence of OAB; the results demonstrated that the odds of OAB-dry occurring in late pregnancy were 0.80 times the odds in early pregnancy (*p* = 0.005). The post hoc polynomial test verified that the trend was linear, at statistical significance (*p* = 0.01), and thus corroborated the finding that OAB-dry is less prevalent in late pregnancy than in early pregnancy; however, middle and late pregnancy did not differ with respect to the prevalence of OAB-dry.

The changes in symptom severity and the degree of symptom distress in OAB during pregnancy indicated that symptoms were significantly (*p* < 0.001) severer—by 0.07 and 0.21 points in middle and late pregnancy, respectively—than in early pregnancy. Furthermore, distress was significantly (*p* < 0.001) greater—by 0.13 and 0.27 points in middle and late pregnancy, respectively—than in early pregnancy. Accordingly, symptom severity and degree of distress were significantly severer in late pregnancy than in early and middle pregnancy. We also used McNemar’s test to analyze the prevalence of OAB between pre-pregnancy and early pregnancy. We found that the prevalence of OAB, OAB-dry, and OAB-wet was significantly higher in early pregnancy than pre-pregnancy (*p* < 0.001).

## 4. Discussion

The results demonstrate that OAB prevalence, symptom severity, and degree of distress increase with the gestational age. For early, middle, and late pregnancy, the prevalence of OAB was 76.9%, 86.3%, and 88.6%, respectively; the prevalence of OAB-dry was 45.4%, 43.3%, and 39.8%, respectively; and the prevalence of OAB-wet was 31.5%, 42.9%, and 48.8%, respectively. OAB and OAB-wet became significantly more prevalent over time, but OAB-dry became significantly less prevalent during late pregnancy. Van Brummen et al. [13] reported that the prevalence of frequent and urgent urination at the 12th week of pregnancy was very high (74% and 63%, respectively), and that the prevalence of OAB was significantly higher at the 36th than the 12th week of pregnancy. Van Brummen et al. [9] further estimated the prevalence of OAB-dry and OAB-wet individually, and reported that at the 12th and 36th weeks of pregnancy, the prevalence of OAB-dry was 46.9% and 46.9%, respectively, while that of OAB-wet was 3.5% and 14.6%, respectively. The prevalence of OAB-dry and OAB-wet reported by van Brummen et al. [9] was consistent with and lower than those obtained by our study, respectively. This difference may be due to our differences in measurement and definitions: van Brummen et al. defined OAB-wet as a disease with the symptoms of frequent urination, urgent urination, and urge urinary incontinence, whereas we defined OAB-wet as a disease with the symptoms of only urgent urination and urge urinary incontinence. Therefore, we observed a higher prevalence because our definition has fewer criteria.

No significant difference was observed in the prevalence of OAB-dry between early and middle pregnancy, although such prevalence was significantly lower during late pregnancy. This may be due to worsening urge urinary incontinence during late pregnancy. Daly, Clarke, and Begley reported that the prevalence of urge urinary incontinence increased from 34.8% before pregnancy to 38.7% after pregnancy [16]. Another study also reported a 32.1–37.2% prevalence of urge urinary incontinence during late pregnancy, where those of urge urinary incontinence and mixed urinary incontinence were 4.8% and 16.8%, respectively [17,18]. This implies that OAB-dry developed into OAB-wet by late pregnancy, which might explain why OAB-wet significantly increases but OAB-dry significantly decreases over time. The preceding result is also partially consistent with that obtained by van Brummen et al. [9]—the prevalence of OAB-dry decreased over time (46.9% at the 12th week of pregnancy and 45% at the third trimester of pregnancy). In our study, pregnancy was divided into three trimesters in order to more precisely track the evolution of OAB’s prevalence during pregnancy. We concluded that the prevalence of OAB-dry significantly increases until middle pregnancy, before decreasing in late pregnancy.

OAB severity and the degree of distress caused by OAB symptoms worsen with the number of weeks of pregnancy. Van Brummen et al. [13] employed a subscale of the urogenital distress inventory (UDI), which measures the self-perceived degree of distress caused by OAB symptoms, with a total score from 0 to 100 points. The mean score increased from 21.6 points at the 12th week to 25.7 points at the 36th week of pregnancy. This study indicates that the degree of distress caused by OAB symptoms becomes severer during pregnancy, which is consistent with our results.

Our study had the following limitations: First, we only recruited participants from a single medical center; hence, the results may not be generalizable to pregnant women from other medical institutions or regions. Furthermore, the participants’ personal factors or other factors might lead to loss of valuable data; therefore, the results cannot apply to participants who withdrew. In addition, bias may have been introduced by the participants retrospectively filling out the items for OAB during pre-pregnancy.

## 5. Conclusions

The prevalence of OAB and OAB-wet increased over the course of pregnancy, but the prevalence of OAB-dry decreased. Furthermore, symptom severity and degree of distress increased over time. These results can inform clinicians and the public with regard to how OAB symptoms change during pregnancy and affect pregnant women. According to our results, clinicians and health care providers may conduct further study in order to identify the proper intervention in early pregnancy to alleviate symptom severity and the impact of OAB on pregnant women.

## Figures and Tables

**Table 1:** Definitions of the urinary symptoms, OAB-dry, and OAB-wet.

Frequency (3a)	Nocturia (4a)	Urgency (5a)	Urge Urinary Incontinence (6a)
≥1 point	≥2 points	≥1 point	≥1 point

OAB-dry: 5a + 3a, 5a + 4a, 5a + 3a + 4a; OAB-wet: OAB-dry + 6a, 5a + 6a.

**Table 2:** Characteristics of participants during early pregnancy.

	Mean ± SD or *n* (%)
Age (years old)	33.82 ± 4.13
Gestational age (weeks)	
Early pregnancy	12.54 ± 2.73
Middle pregnancy	23.90 ± 3.28
Late pregnancy	33.61 ± 2.74
Parity	
Nulliparous	430 (65.3)
1	191 (29.0)
≥2	38 (5.7)
Number of previous deliveries	
Vaginal delivery	
0	508 (77.1)
1	131 (19.9)
≥2	20 (3.0)
Caesarean section	
0	572 (86.8)
1	76 (11.5)
≥2	11 (1.7)

SD: standard deviation.

**Table 3:** Changes in the prevalence of OAB.

	Beta	SE	Exp(B)	95% Confidence Interval		*p*-Value
				Lower	Upper	
	**OAB**					
Early			ref.			
Middle	0.64	0.11	1.90	1.53	2.36	<0.001 **
Late	0.85	0.12	2.33	1.84	2.97	<0.001 **
	**OAB-dry**					
Early			ref.			
Middle	−0.08	0.08	0.92	0.79	1.08	0.32
Late	−0.23	0.08	0.80	0.68	0.93	0.005 *
	**OAB-wet**					
Early			ref.			
Middle	0.49	0.08	1.63	1.41	1.89	<0.001 **
Late	0.73	0.08	2.07	1.78	2.41	<0.001 **
	**Symptom severity**					
Early	ref.					
Middle	0.07	0.02	--	0.03	0.10	<0.001 **
Late	0.21	0.02	--	0.17	0.24	<0.001 **
	**Symptom distress**					
Early	ref.					
Middle	0.13	0.03	--	0.07	0.19	<0.001 **
Late	0.27	0.03	--	0.21	0.33	<0.001 **

OAB: overactive bladder; SE: standard error; ref: reference. Significance: * *p* < 0.01; ** *p* < 0.001.

## Data Availability

The data that support the findings of this study are available from the corresponding author upon reasonable request.
